# Draft Genome Sequence of *Burkholderia contaminans* 293K04B, an Endosymbiont of the Sponge-Derived Fungus *Stachylidium bicolor*

**DOI:** 10.1128/genomeA.01142-17

**Published:** 2017-11-09

**Authors:** Katja M. Fisch, Cristina Silva Pereira, Olga Genilloud, Celso Almeida, Till F. Schäberle

**Affiliations:** a Institute for Insect Biotechnology, Justus Liebig University Giessen, Giessen, Germany; b Instituto de Tecnologia Química e Biológica António Xavier, Universidade Nova de Lisboa, ITQB NOVA, Oeiras, Portugal; c Fundación MEDINA, Parque Tecnológico de Ciencias de la Salud, Armilla, Granada, Spain; d German Centre for Infection Research (DZIF) Partner Site Bonn/Cologne, Bonn, Germany

## Abstract

Here, we present the draft genome of the endofungal symbiotic bacterium *Burkholderia contaminans* 293K04B, isolated from *Stachylidium bicolor* 293K04 (Ascomycota). The fungus was originally isolated from the sponge *Callyspongia* cf. *C. flammea*. *S. bicolor* 293K04 produces the endolides A-B, bioactive cyclic peptides possibly biosynthesized by its endobacterium *B. contaminans* 293K04B.

## GENOME ANNOUNCEMENT

Species of the genus *Burkholderia* are widely distributed in diverse habitats and are known as human and plant pathogens, plant growth promoters, and endosymbionts (1). Some defined bacterial endosymbionts have been recognized as true producers of secondary metabolites that were originally isolated from their hosts (2). Until recently, bacterial endosymbionts have rarely been seen in fungal hosts (3). The first secondary metabolites discovered to be produced by endofungal bacteria were the antimitotic rhizoxins, initially isolated from *Rhizopus microsporus*, and later found to be produced by its endosymbiont *Burkholderia rhizoxinica* (4). The heptapeptidic rhizonins contain units of the rare amino acid 3-(3-furyl)-alanine. These compounds were also isolated from cultures of a different *R. microsporus* strain, yet were later found to be produced by its endosymbiont *Burkholderia endofungorum* (5). The tetrapeptides endolides A and B, also comprising 3-(3-furyl)-alanine units, have been isolated from the marine-derived fungus *S. bicolor* 293K04. These compounds showed interaction with vasopressin and serotonin receptors (6). An endosymbiotic bacterial strain was successfully isolated from mycelia exposed to mechanical shearing. This strain belongs to the *Burkholderia* genus, and accurate phylogenetic placement of the strain was performed by multilocus sequence analysis (MLSA), further corroborated by *in silico* DNA-DNA hybridization experiments (Almeida C, Silva Pereira C, Gonzalez-Menendez V, Bills G, Pascual J, Sánchez-Hidalgo M, Kehraus S, and Genilloud O, unpublished data), which revealed it to be a member of the species *B. contaminans*. Hence, the bacterium was named *B. contaminans* 293K04B. Disclosing its genome may provide valuable insight for understanding the biology of its symbiotic relationship with *S. bicolor* 293K04, especially its role in the biosynthesis of endolides as a putative symbiotic function of the endobacteria.

DNA of *B. contaminans* 293K04B was isolated from a 2-day-old culture grown in liquid trypticase soy broth (TSB) medium. Genomic DNA was extracted according to the cetyltrimethylammonium bromide/polyvinylpyrrolidone (CTAB/PVP) protocol (7). Purification was achieved using the Purelink Genomic DNA minikit (Invitrogen) and yielded 100 ng/µL concentration with an OD_260/280_ ratio of 1.86. Two sets of Illumina paired-end data were assembled using SPAdes, resulting in a draft genome sequence consisting of 100 contigs with a minimum size of 541 bp. The genome size of *Burkholderia* species is very variable from 2.4 Mb (“*Candidatus* Burkholderia schumannianae” UZHbot8) (8) to more than 10 Mb, e.g., 11.5 Mb in the case of *Burkholderia terrae* BS001 (9). The genome of *B. contaminans* 293K04B measures 8.8 Mb. AntiSMASH (10) analysis revealed 16 biosynthetic gene clusters (BGCs) for the production of specialized metabolites. One BGC contains a polyketide synthase (PKS), 2 BGCs contain nonribosomal peptide synthetase (NRPS), and 1 BGC shows a hybrid PKS-NRPS.

### Accession number(s).

This draft genome has been deposited at DDBJ/EMBL/GenBank under the accession number NQOD00000000. The version described in this paper is the first version, NQOD01000000.
